# Neuropsychiatric Sequelae Following ACA Aneurysm Repair: A Case Report and Review

**DOI:** 10.1192/j.eurpsy.2026.11054

**Published:** 2026-07-31

**Authors:** L. M. Kebede, T. Rasul, H. Raai

**Affiliations:** Psychiatry, SBH Health System, New York City, United States

## Abstract

**Introduction:**

Neuropsychiatric manifestation such as behavioral disinhibition, paranoia and cognitive changes can be a sequela of Anterior Cerbral Artery aneurysm and repair^1,2^. Clinical presentation can pose significant diagnostic and management challenges and often requires a multidisciplinary approach.

**Objectives:**

To describe a complex case of post-intracranial hemorrage neuropsychiatric symptoms and discuss diagnostic considerations and management strategies at the junction of neurology and psychiatry.

**Methods:**

Case report and literature review

**Results:**

We present the case of a 67-year-old man with a history of hypertension and ICH status-post interventional radiology aneurysm embolization, complicated by aneurysm re-rupture, balloon-assisted coiling with Integrilin, and severe vasospasm requiring verapamil and neuro-ICU admission. Following which patient developed disorientation, paranoia, and aggressive behavior. He presented to the emergency department after eloping from another hospital and was found wandering. The patient presented disorganized and anxious, with tangential thought processes, and labile affect. Collateral history indicated ongoing intermittent disorientation, paranoid delusions and aggression since neurosurgical intervention.

**Conclusions:**

This case highlights the complex neuropsychiatric sequelae that can arise after intracranial hemorrage and aneurysm repair. We review the literature on post- Anterior Cerbral Artery aneurysm cognitive and behavioral syndromes, discuss the role of consultation-liaison psychiatry in differentiating neuropsychiatric conditions from primary psychiatric disorders, and consider management strategies for agitation and psychosis in medically fragile patients.

**Disclosure of Interest:**

None Declared

